# A comprehensive, longitudinal analysis of humoral responses specific to four recombinant antigens of SARS-CoV-2 in severe and non-severe COVID-19 patients

**DOI:** 10.1371/journal.ppat.1008796

**Published:** 2020-09-10

**Authors:** Yuxin Chen, Xin Tong, Yang Li, Bin Gu, Jiawei Yan, Yong Liu, Han Shen, Rui Huang, Chao Wu

**Affiliations:** 1 Department of Laboratory Medicine, Nanjing Drum Tower Hospital, Nanjing University Medical School, Nanjing, Jiangsu, China; 2 Department of Infectious Diseases, Nanjing Drum Tower Hospital, Nanjing University Medical School, Nanjing, Jiangsu, China; 3 Medical Technology School of Xuzhou Medical University, Xuzhou Key Laboratory of Laboratory Diagnostics, Xuzhou, Jiangsu, China; 4 Department of Laboratory Medicine, the Affiliated Hospital of Xuzhou Medical University, Xuzhou, Jiangsu, China; 5 Department of Laboratory Medicine, Xuzhou Infectious Disease Hospital of Xuzhou Medical University, Xuzhou, Jiangsu, China; 6 Department of Experimental Medicine, Nanjing Drum Tower Hospital, Nanjing University Medical School, Nanjing, Jiangsu, China; Erasmus Medical Center, NETHERLANDS

## Abstract

There is an urgent need for effective treatment and preventive vaccine to contain this devastating global pandemic, which requires a comprehensive understanding of humoral responses specific to SARS-CoV-2 during the disease progression and convalescent phase of COVID-19 patients. We continuously monitored the serum IgM and IgG responses specific to four SARS-CoV-2 related antigens, including the nucleoprotein (NP), receptor binding domain (RBD), S1 protein, and ectodomain (ECD) of the spike protein among non-severe and severe COVID-19 patients for seven weeks since disease onset. Most patients generated humoral responses against NP and spike protein-related antigens but with their distinct kinetics profiles. Combined detection of NP and ECD antigens as detecting antigen synergistically improved the sensitivity of the serological assay, compared to that of using NP or RBD as detection antigen. 80.7% of convalescent sera from COVID-19 patients revealed that the varying extents of neutralization activities against SARS-CoV-2. S1-specific and ECD-specific IgA responses were strongly correlated with the neutralization activities in non-severe patients, but not in severe patients. Moreover, the neutralizing activities of the convalescent sera were shown to significantly decline during the period between 21 days to 28 days after hospital discharge, accompanied by a substantial drop in RBD-specific IgA response. Our data provide evidence that are crucial for serological testing, antibody-based intervention, and vaccine design of COVID-19.

## Author summary

The world is facing an unprecedented challenge with communities and economies affected by the growing pandemic of coronavirus disease 2019 (COVID-19). Currently, there is no vaccine or effective drugs have been approved to treat or prevent COVID-19. The development of antibody response to SARS-CoV-2, the virus that causes COVID-19, started to be reported but remained largely elusive. Understanding the adaptive responses where the body makes antibodies that specifically bind to the SARS-CoV-2 among COVID-19 patients provides fundamental information for developing effective treatment and preventive vaccine. In this study, we not only successively analyzed the specificity and magnitude of antibody responses using four SARS-CoV-2 related antigens, but also monitored the neutralization potency of the convalescent sera from COVID-19 patients at the time point of hospital discharge and follow-up visit. Our results indicated that most patients generated humoral responses against nucleoprotein and three spike protein-related antigens with their distinct kinetics profiles. Additionally, most convalescent sera had the varying extents of neutralization activities against SARS-CoV-2. Of note, we identified that IgA antibody responses specific to S1 and ECD were strongly correlated with neutralization activities in non-severe patients, but not in severe patients. Furthermore, we identified a significant reduction of neutralizing activities of the convalescent sera within one month. Our data provide a collective basis of serological testing, antibody-based intervention, and vaccine design of COVID-19.

## Introduction

The ongoing pandemic of severe acute respiratory syndrome coronavirus 2 (SARS-CoV-2) that first emerged in China, has rapidly spread worldwide. As of June 29, 2020, the coronavirus disease 2019 (COVID-19) had been confirmed in more than 10 million cases worldwide [1], carrying a mortality rate of 1.3% [2]. There is an urgent need for effective treatment and preventive vaccine to contain this devastating global pandemic, which requires a comprehensive understanding of humoral responses specific to SARS-CoV-2 during the disease progression and convalescent phase of COVID-19 patients.

We recently reported that differential longitudinal patterns of nucleic acid and serology testing among severe patients, non-severe patients, and asymptomatic carriers of SARS-CoV-2 [3]. We demonstrated that early, arising antibody responses were detected concurrent with positive viral RNA among severe patients, while antibody responses which often facilitate the viral clearance were observed from non-severe patients. This prompted us to further explore the antigen specificity and humoral responses among severe and non-severe patients. The kinetics of antibody response to nucleoprotein (NP) and spike protein receptor binding domain (RBD) from SARS-CoV-2 have been reported [4, 5]. However, in addition to RBD, the antibody response targeting the full-length S1 domain or ectodomain (ECD) of the spike protein is still not known. The RBD of SARS-CoV-2 is relatively small containing 222 amino acids, compared to the full-length S1 protein or ectodomain (ECD) of the spike glycoprotein. Therefore, the sensitivity of antibody seroconversion might be compromised by exclusively including the RBD instead of alternative recombinant antigens such as S1 and ECD protein for COVID-19 serological testing. Additionally, recent studies demonstrated that transfusion of convalescent plasma containing the neutralizing antibodies resulted in clinical improvement [6, 7], which necessitates a comprehensive understanding of the specificity, isotypes, potency and persistence of the neutralizing antibody components present in the convalescent sera of clinically recovered COVID-19 patients.

In this retrospective study, we successively monitored the serum IgM and IgG responses specific to four SARS-CoV-2 related antigens, including the NP protein, RBD protein, S1 protein, and ECD protein in 19 non-severe and 7 severe COVID-19 patients during the disease progression. Moreover, the neutralization activities of the serum collected at hospital discharge were determined, and their correlations with the specific antibody of different isotypes targeting the above four recombinant antigens were identified. Furthermore, in order to determine the duration of antibody responses, the neutralization activities and anti-SARS-CoV-2 antibody responses after hospital discharge were also analyzed. Our data provide important insights for serological testing, antibody-based intervention, and vaccine design.

## Materials and methods

### Expression and purification of four SARS-CoV-2 related proteins

**Fig 1:**
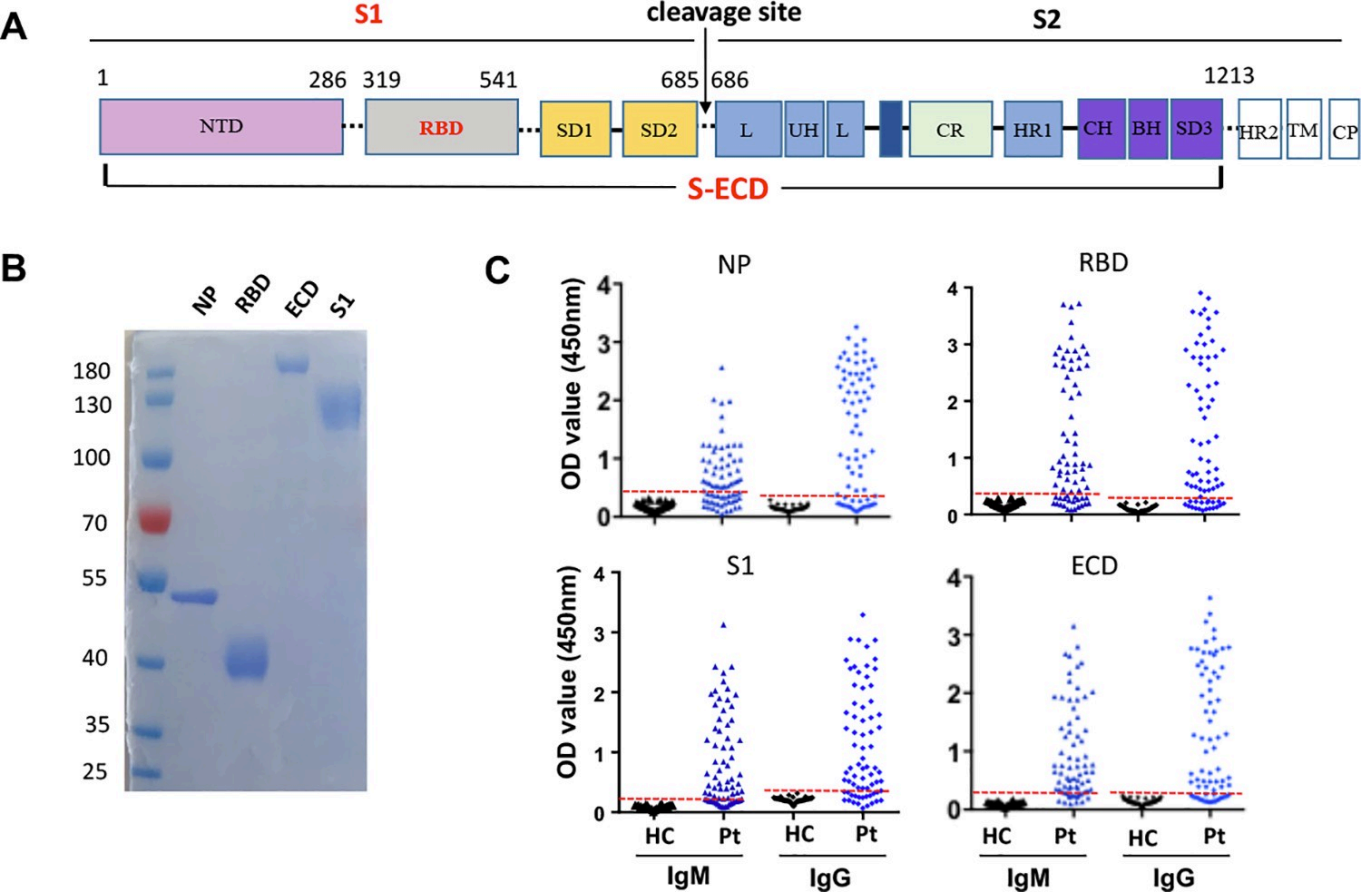
Establishment of the serological assay for SARS-CoV-2. **(A)** Schematic representation of RBD, S1, and ectodomain (ECD) of the SARS-CoV-2 spike protein. **(B)** The expression and purification of RBD, S1, ECD, and NP protein. **(C)** ELISA assay for detecting IgM and IgG humoral responses against RBD, S1, ECD and NP protein was performed using sera samples diluted at 1:200. The dashed blue line indicates the cutoff value for each assay, which was determined based on the archived serum samples collected before the COVID-19 outbreak. Sera from 46 healthy controls (HC) collected in 2019 served as the negative control, whereas 72 samples collected at different time points from COVID-19 patients were used as the positive control.

Four SARS-CoV-2 related proteins, recombinant spike protein receptor binding domain (RBD) protein, S1 protein, the ectodomain of the spike protein (ECD), and nucleocapsid protein (NP) were used as detected antigens, respectively **(Fig 1A).** The genes encoding spike RBD (residues Arg319 to Phe541 of spike protein), S1 protein (residue Val20 to Arg685 of spike protein), the ectodomain of spike protein (ECD) (residue Met1 to Tyr1213 of spike protein), and the full-length NP protein were codon-optimized and synthesized (Genewiz, China). Genes encoding for the spike RBD, S1, and ECD were cloned into the mammalian expression vector pcDNA3.4, while the genes encoding the NP protein were inserted into the expression vector pET-28(b), in frame respectively and upstream of the series of six histidine residues. The RBD, S1, and ECD proteins were expressed in 293f cells, while the NP protein was from *Escherichia coli*, followed by affinity purification. The purity of the NP protein and RBD proteins was determined by 10% sodium dodecyl sulfate (SDS) polyacrylamide gel electrophoresis **(Fig 1B)**.

### Ethic statement

This study was approved by ethics committee of Affiliated Hospital of Xuzhou Medical University and Nanjing Drum Tower Hospital in Jiangsu Province (Protocol number: XYFY2020-KL016-01 and 2020-023-01). Written informed consent was waived by the Ethics Commission due to public health outbreak investigation.

### Patients and clinical specimens

We retrospectively recruited 26 patients with COVID-19 from Jan 30 to Feb 25, 2020, at the Affiliated Hospital of Xuzhou Medical University and Nanjing Drum Tower Hospital in Jiangsu Province. Patients with suspected SARS-CoV-2 were confirmed after two sequential positive respiratory tract sample results. Throat swab samples were collected every 1–2 days. The serum samples retrieved from routine biochemical or immunological testing were inactivated at 56°C for 30 min. These samples were later stored at -80°C for later serological detection.

The severity of COVID-19 was judged based on the sixth revised trial version of novel coronavirus pneumonia diagnosis and treatment guidance [8]. Those who met the criterion as follows were defined as severe cases: (1) Respiratory distress with respiratory rate over 30 per minute; (2) Hypoxia (oxygen saturation less than ≤93% in the resting state); (3) Arterial blood oxygen partial pressure (PaO2)/oxygen concentration (FiO2) less than 300 mm Hg; or (4) Severe disease complications including respiratory failure which requires mechanical ventilation, septic shock, or non-respiratory organ failure.

### Enzyme-linked immunosorbent assay (ELISA)

An in-house enzyme-linked immunosorbent assay (ELISA) was performed as previously described (8). Briefly, 96-well plates were coated with 500 ng/mL of individual recombinant viral antigen overnight, respectively. The plates were incubated with serum samples in a dilution of 1:200, followed by incubation with either anti-human IgM conjugated with HRP (Abcam, ab97205, 1:2000 dilution) and IgG conjugated with HRP (Abcam, ab6759, 1:100000 dilution). Optical density (OD) value at 450nm was measured. For antibody isotyping of the sera collected during convalescent COVID-19 patients, the plates were firstly coated with the desired recombinant antigen, respectively. Later, the plates were added with serial diluted serum samples starting from 1:200 to 1:437400. After washing, the plates were coated with anti-human IgA conjugated with HRP (Abcam, ab97215, 1:1000 dilution), anti-human IgG1 conjugated with HRP (Abcam, ab99774, 1:2000 dilution), anti-human IgG2 conjugated with HRP (Abcam, ab99779, 1:1000 dilution), anti-human IgG3 conjugated with HRP (Abcam, ab86252, 1:2000 dilution), and anti-human IgG4 conjugated with HRP (Abcam, ab99823, 1:1000 dilution) for antibody isotyping analyses. Subsequently, the plates were incubated with TMB substrate for 1 hour and the reaction stopped with 1M H_2_SO_4_. The preliminary cut-off value for each ELISA assay was calculated as the mean of the negative control serum OD values plus 2 standard deviation (SD) from 45 archived healthy individuals from the year of 2019. Antibody endpoint titer was determined by the highest dilution which gives an OD value higher than cut off value of the healthy control group at the same dilution.

### Microneutralization assay

Pseudoviruses expressing the SARS-CoV-2 spike protein were obtained as a general gift from the institute of biological product control from National institute for Food and Drug Control of China. SARS-CoV-2 pseudoviruses were prepared using VSV G pseudotyped viruses (G*ΔG-VSV) that package the expression cassette for firefly luciferase instead of VSV-G in the VSV genome, and the serum neutralization capability was performed as described recently [9]. Briefly, the SARS-CoV-2 pseudoviruses were preincubated with heat-inactivated serum samples at a 3-fold serial dilution starting at 1:20 at 37°C for one hour, together with the pseudovirus control and cell control wells. The sera from healthy controls were served as the negative control in hexaplicate. Next, the 96-well plates were seeded with 100 μL of freshly trypsinized Huh7 cells (2x10^4^ cells/well). After 24 hours of incubation in a 5% CO_2_ environment at 37°C, the luminescence was measured using luciferase substrate (Promega, E6120). The titer of NAbs was calculated as 50% inhibitory dose (ID50), expressed as the reciprocal serum dilution which resulted in a 50% reduction in relative light units (RLUs) compared to the virus control wells after subtraction of background RLUs.

### Data collection

Data including demographic information, medical history, symptoms, laboratory findings, treatment regimen were retrieved from the patients' medical record. Laboratory results included blood routine, lymphocyte subsets, and CRP. The total number of lymphocytes in peripheral blood was counted by hemocytometer. The percentage of lymphocyte subsets were analyzed with FACS flow cytometry for those COVID-19 patients on admission.

### Statistical analysis

Binding antibody titers or neutralizing antibody titers were expressed as geometric mean titers (GMTs). The mean (standard deviation (SD)) or median (interquartile range (IQR)) was used to present the continuous variables. Categorical variables were described as the count and percentage. The independent group t test (normal distribution) and Mann-Whitney U (non-normal distribution) were used to compare continuous variables between groups. SPSS software program version 22.0 (Chicago, IL, USA) were used for data analysis. p<0.05 was considered to be statistically significant. When multiple testing was performed in the correlation analysis, the Bonferroni correction adjusted significance level was 0.05/3 = 0.016.

## Results

### Demographic and clinical characteristics of COVID-19 patients in our cohort

A total of 26 COVID-19 patients were enrolled in our study **(Table 1 and S1 Table).** The median age was 47 years (IQR, 35.5–57.5), and 14 patients were male. The median age for severe COVID-19 patients was significantly higher compared to the non-severe group (p = 0.005). Type 2 diabetes (42.9%) was the most common comorbidities. Fever (85.7%), cough (100%), and myalgia (28.6%) were the most common symptoms. Blood test on admission revealed that the lymphocyte counts were significantly lower in the severe cases (0.86 x 10^9^/L) than in the non-severe cases (1.36 x 10^9^/L) (p<0.001). Lymphocyte subset analyses further indicated that the numbers of T lymphocytes, CD8+ T cells and B cells were markedly reduced in severe patients, in comparison to non-severe patients (p<0.001, p = 0.005, p = 0.007, respectively) (**S1 Table**). All the severe and non-severe patients were administered with empirical antimicrobial treatments including interferon α inhalation, arbidol, lopinavir-ritonavir combination and darunavir. As of March 25th, 2020, the 26 patients were clinically recovered from COVID-19 and subsequently discharged.

**Table 1:** Demographic and epidemiologic characteristics of severe and non-severe COVID-19 patients.

Variables (n [%] or median [IQR])	All patients (n = 26)	severe (n = 7)	Non-severe (n = 19)	p value*
Age (ys)	47 (35.5, 57.5)	56 (49.5, 62.0)	41 (31, 56)	0.006
Sex				0.665
Female	12 (46.2)	4 (57.1)	8 (42.1)	
Male	14 (53.8)	3 (42.9)	11 (57.9)	
**Onset signs and symptoms**				
Cough	17 (65.4)	7 (100)	10 (52.6)	0.058
Fever	15 (57.5)	6 (85.7)	9 (47.4)	0.178
Myalgia	3 (11.5)	2 (28.6)	1 (5.3)	0.167
Shortness of breath	2 (7.7)	0 (0)	2 (10.5)	1.000
**Comorbidities**				
Diabetes	4 (15.4)	3 (42.9)	1 (5.3)	0.047
Hypertension	2 (7.7)	1 (14.3)	1 (5.3)	1.000
Alzheimer's disease	1 (3.8)	1 (14.3)	0 (0)	0.269
Cerebral infarction	1 (3.8)	1 (14.3)	0 (0)	0.269
Liver cyst	1 (3.8)	0 (0)	1 (5.3)	1.000
The duration of positive viral RNA (days)	18.0 (10.0, 25.0)	19.0 (13.5, 26.0)	17.5 (7.8, 23.8)	0.149
ct value of RT-PCR	27.0 (24.0, 35.0)	24 (23.5, 28.5)	28.5 (24.0, 35.0)	0.038
Lymphocyte (×10^9^/L)	1.2 (0.9, 1.8)	0.9 (0.7, 1.1)	1.4 (1.1, 1.9)	<0.001
CRP (mg/L)	26.4 (19.8, 39.1)	31.0 (22.0, 35.0)	23.3 (20.5, 39.1)	0.324
ESR (mm/h)	15.5 (6.3, 34.3)	21.0 (12.0, 39.0)	10.0 (5.5, 30.0)	0.333
IL-6 (pg/ml)	0.013 (0.003, 0.022)	0.02 (0.0095, 0.0265)	0.08 (0.0028, 0.0165)	0.308

IQR, interquartile range; ct, cycle threshold; CRP, C-reactive protein; ESR, erythrocyte sedimentation rate; IL-6, Interleukin- 6.

*p value refers to the statistical difference between severe and non-severe group.

### Dynamic and distinct anti-SARS-CoV-2 IgM and IgG antibody profile targeting NP protein and three spike protein-related antigens

**Fig 2:**
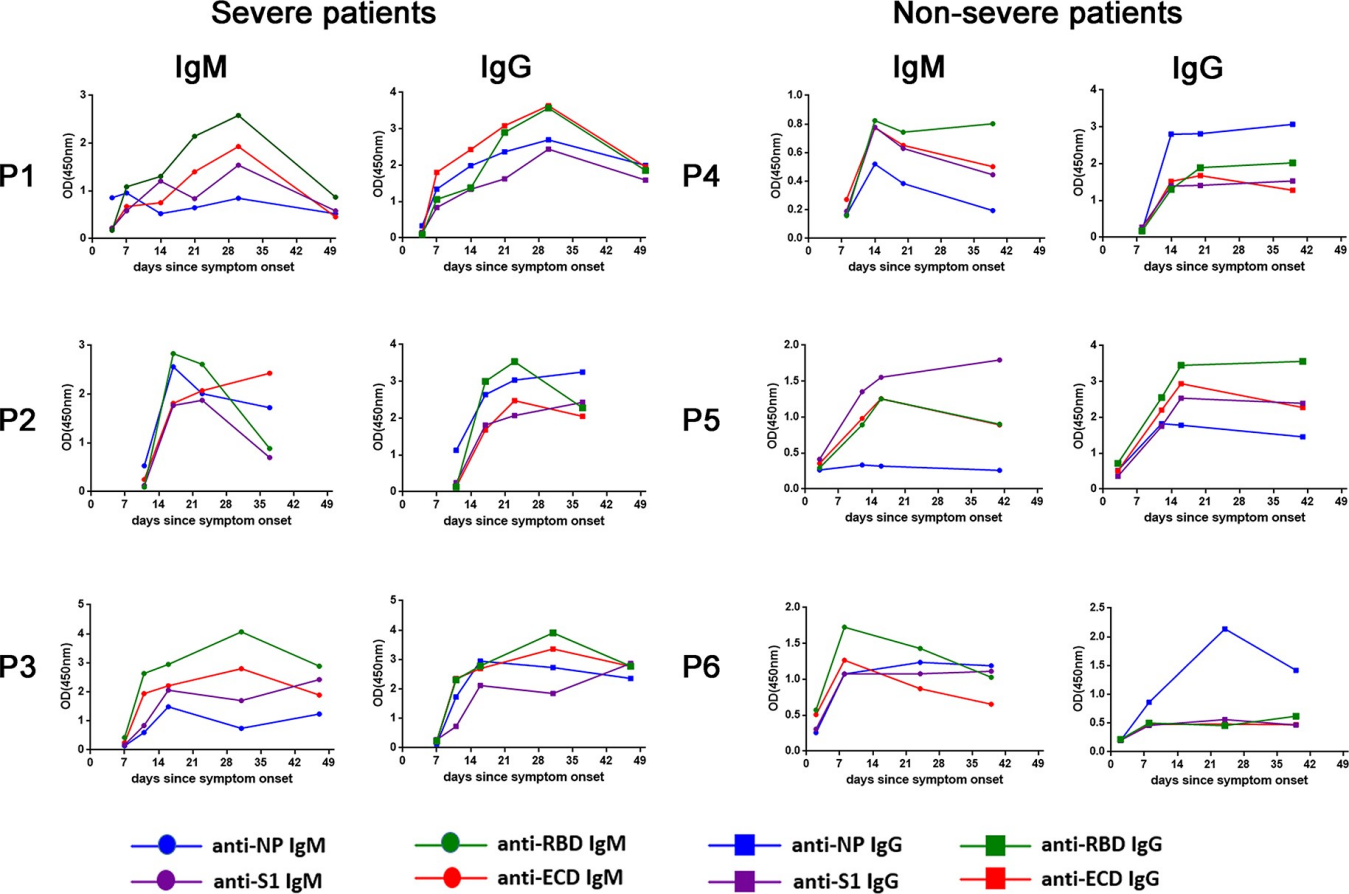
Representative dynamic IgM or IgG responses specific to NP, RBD, S1, and ECD for three severe COVID-19 patients and three non-severe COVID-19 patients over time since symptom onset, determined by ELISA assay.

108 serum specimens were obtained from 26 COVID-19 patients (mean 4.1 serum samples per patient). We serially monitored the antibody profile specific to RBD, S1, ECD and NP during the disease progression phase of COVID-19 patients **(Fig 2 and S1 Fig).** Overall, 24 (92.31%) out of 26 patients were seroconverted, whereas 2 patients (P22 and P23) showed a barely detectable level of both IgM and IgG response against four SARS-CoV-2 antigens during our observation period. We cannot rule out the possibility that these two patients might have been seroconverted later after hospital discharge. An increase in serum IgM and IgG antibody levels against four proteins for most patients at 4 days or later after symptom onset was noticed, as illustrated by the OD values in **Fig** 2. Throughout our observation period, 80.7% (21/26) of patients exhibited a strong response of IgM or IgG against four antigens, while three patients (P13, P17 and P18) had a relatively low level of antibody response against spike protein derived antigens. Furthermore, distinct kinetics of antibodies with different specificities were identified. The patients initially reached the NP-specific IgM peak and S1-specific IgM peak on day 21 (IQR: 16–25 and IQR: 16–30, respectively), then anti-RBD IgM, anti-ECD IgM, and anti-NP IgG peaked at day 25 (IQR: 21–28, IQR: 21–30, IQR: 18–34, respectively), followed by the RBD-specific IgG peaked on day 28 (IQR:23–34), and the peak IgG responses specific of S1 and ECD antigens on day 33 (IQR: 21–40, IQR:28–41, respectively). Interestingly, antibody responses were sharply dropped in 25% (6/24) of patients after reaching their peak, whereas the antibody responses in the remaining patients were sustained. Our data suggested that SARS-CoV-2 infection induced a distinct temporal profile of humoral responses against viral NP, RBD, S1 and ECD antigens.

### Correlation analyses of antibody responses targeting RBD, S1, ECD, and NP

**Fig 3:**
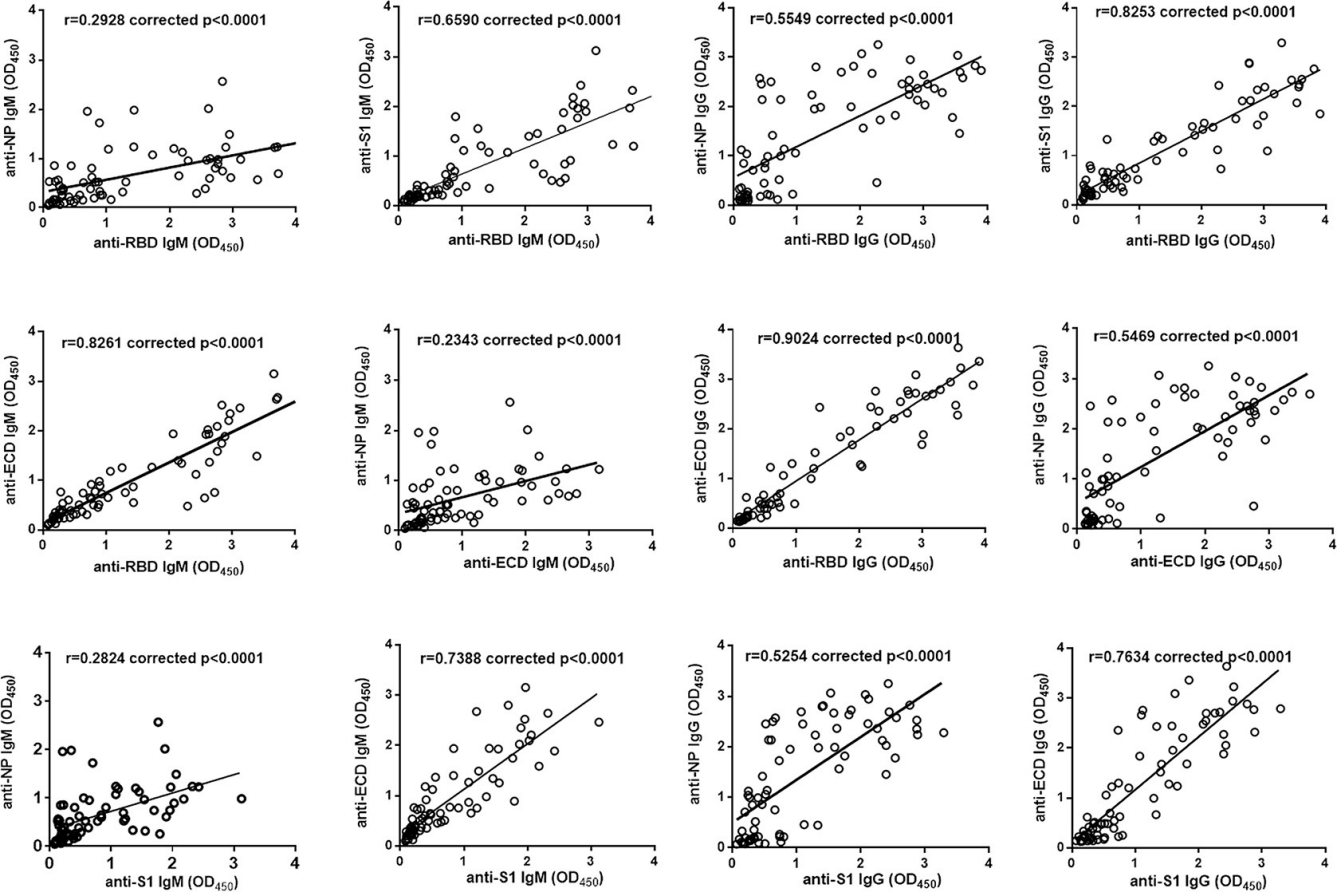
Correlation analysis of IgM or IgG responses specific to NP, RBD, S1, and ECD. The Bonferroni correction was used and the adjusted significance level of p value was 0.05/3 = 0.016.

Correlation analyses of OD values derived from four ELISA assays were performed (**Fig 3**). The anti-RBD IgM response was correlated with anti-NP IgM (r = 0.2928, corrected p<0.0001), anti-S1 IgM (r = 0.6590, corrected p<0.0001), and anti-ECD IgM (r = 0.8261, corrected p<0.0001), while the anti-RBD IgG response was associated with the levels of anti-NP IgG (r = 0.5549, corrected p<0.001), anti-S1 IgG (r = 0.8253, corrected p<0.0001), and anti-ECD IgG responses (r = 0.9024, corrected p<0.0001). Besides, the anti-S1 IgM response was associated with the anti-N IgG response (r = 0.2824, corrected p<0.0001) and anti-ECD IgM responses (r = 0.7338, corrected p<0.0001). The anti-S1 IgG response was linked with the anti-NP IgG (r = 0.5254, corrected p<0.0001) and anti-ECD IgG response (r = 0.7634, corrected p<0.0001), anti-ECD IgM response was correlated with the anti-NP IgM response (r = 0.2343, corrected p<0.0001), whereas anti-ECD IgG response was correlated with anti-N IgG response (r = 0.5469, corrected p<0.0001). Overall, the level of RBD-specific IgM or IgG was well correlated with either the anti-S1 or anti-ECD antibody responses, while the NP-specific IgM or IgG was weakly related with the level of IgM or IgG response targeting various subunits of the spike protein. Our data indicated a distinct recognition pattern of NP protein and various domains of spike protein in the serum of COVID-19 patients.

### Combined ECD-specific IgM and NP-specific IgG improved the sensitivity of COVID-19 serological testing at an early infection phase

Since the temporal profiles of NP-specific and spike protein-specific antibody responses were distinct, we determined the sensitivity of serological testing using different SARS-CoV-2 derived antigens from week 1 to week 4 after disease onset. Since the number of sera collected after week 4 of disease onset was relatively limited in our cohort, the sensitivity of our ELISA assay with the clinical samples within the first four weeks was analyzed. As shown in **Table 2**, among the eight anti-SARS-CoV-2 specific IgM or IgG parameters, serological testing of NP protein-specific IgG was able of achieving optimal sensitivities of 36.36%, 75.00%, 89.47%, and 83.33% from week 1 to week 4 after disease onset, respectively. However, the combined testing of ECD-specific IgM and NP-specific IgG responses were able to further synergistically boost the assay sensitivity to 68.18%, 95.83%, 100%, and 83.33% from week 1 to week 4, respectively, yielding the highest sensitivity if compared to that obtained using either combined anti-RBD IgM and anti-RBD IgG or combined anti-NP IgM and anti-NP IgG.

**Table 2:** Detection sensitivity of serological analysis using four SARS-CoV-2 related antigens at different time since disease onset.

Days after onset	1–7		8–14		15–21		22–28		29–42		after 42	
number of samples	22		24		19		12		16		15	
antibody specificity	N (+)	Sensitivity (%)	N (+)	Sensitivity (%)	N (+)	Sensitivity (%)	N (+)	Sensitivity (%)	N (+)	Sensitivity (%)	N (+)	Sensitivity (%)
RBD IgM	4	18.18	12	50.00	19	100.00	10	83.33	15	93.75	13	86.67
S1 IgM	6	27.27	17	70.83	18	93.75	10	83.33	15	93.75	14	93.33
ECD IgM	8	36.36	18	75.00	19	100.00	10	83.33	15	93.75	15	100.00
NP IgM	6	27.27	16	66.67	13	68.42	7	58.33	12	75.00	12	80.00
RBD IgG	6	27.27	12	50.00	17	89.47	9	75.00	14	87.50	13	86.67
ECD IgM	5	22.73	13	54.17	18	93.75	10	83.33	14	87.50	14	93.33
S1 IgM	6	27.27	15	62.5	19	89.47	9	75.00	12	75.00	14	93.33
NP IgM	8	36.36	18	75.00	17	89.47	10	83.33	15	93.75	15	100.00
NP IgG + ECD IgM	15	68.18	23	95.83	16	100.00	10	83.33	15	93.75	15	100.00
NP IgG + RBD IgM	11	50.00	18	75.00	19	100.00	10	83.33	15	93.75	15	100.00
NP IgG + NP IgM	9	40.91	19	79.17	18	93.75	10	83.33	15	93.75	15	100.00
NP IgG + S1 IgM	12	54.55	22	91.67	18	93.75	10	83.33	15	93.75	15	100.00
RBD IgM + RBD IgG	8	36.36	18	75.00	19	93.75	10					83.33	15	93.75	13	86.67

### Higher magnitude of anti-RBD IgG and IgA responses observed in the convalescent serum of severe patients than in the non-severe patients

**Fig 4:**
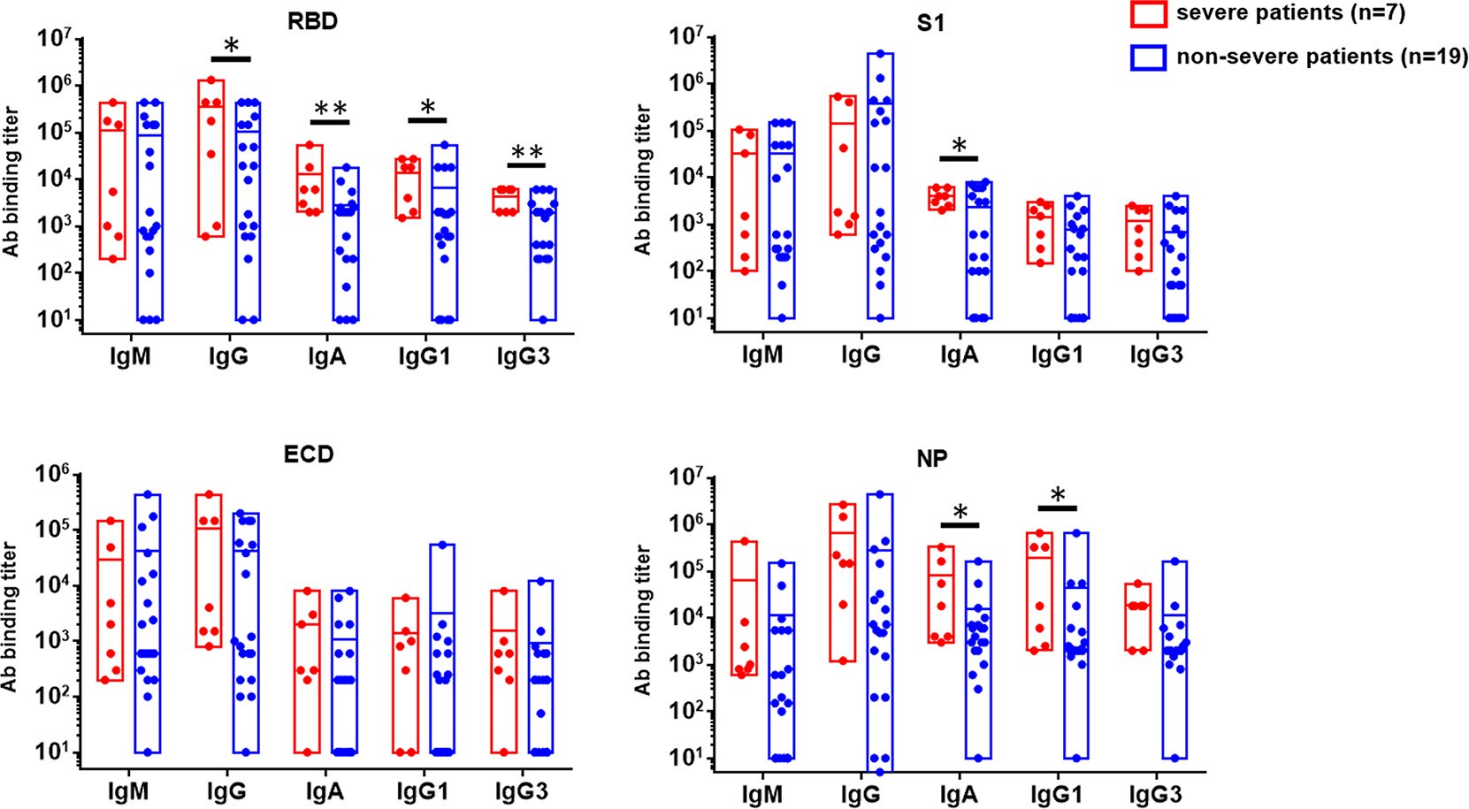
The IgM, IgG, IgA, IgG1, and IgG3 binding tiers specific with RBD, S1, ECD, and NP, respectively, in the convalescent sera of non-severe and severe COVID-19 patients.

To dissect the antibody component and specificity from the sera of COVID-19 clinically recovered patients, the magnitude of IgM, IgA, IgG, and IgG isotypes including IgG1 to IgG4 targeting RBD, S1, ECD, and NP were further analyzed by ELISA **(Fig 4).** Of note, barely detectable levels of anti-SARS-CoV-2 IgG2 and IgG4 were observed from all the patients in our cohort. Compared to non-severe patients, severe patients tended to generate a higher level of antibody responses. Specifically, remarkably higher level of RBD-specific IgG, IgA, IgG1, and IgG3 titers were found in severe patients compared to non-severe patients (p = 0.018, p = 0.009, p = 0.043, p = 0.004, respectively). Likewise, considerably higher levels of S1-specific IgA, NP-specific IgA and IgG1 were also present in severe patients when compared to non-severe patients (p = 0.020, p = 0.012, and p = 0.027, respectively).

### The neutralizing activities of convalescent sera strongly correlated with S1-specific and ECD-specific IgA responses in non-severe patients

**Fig 5:**
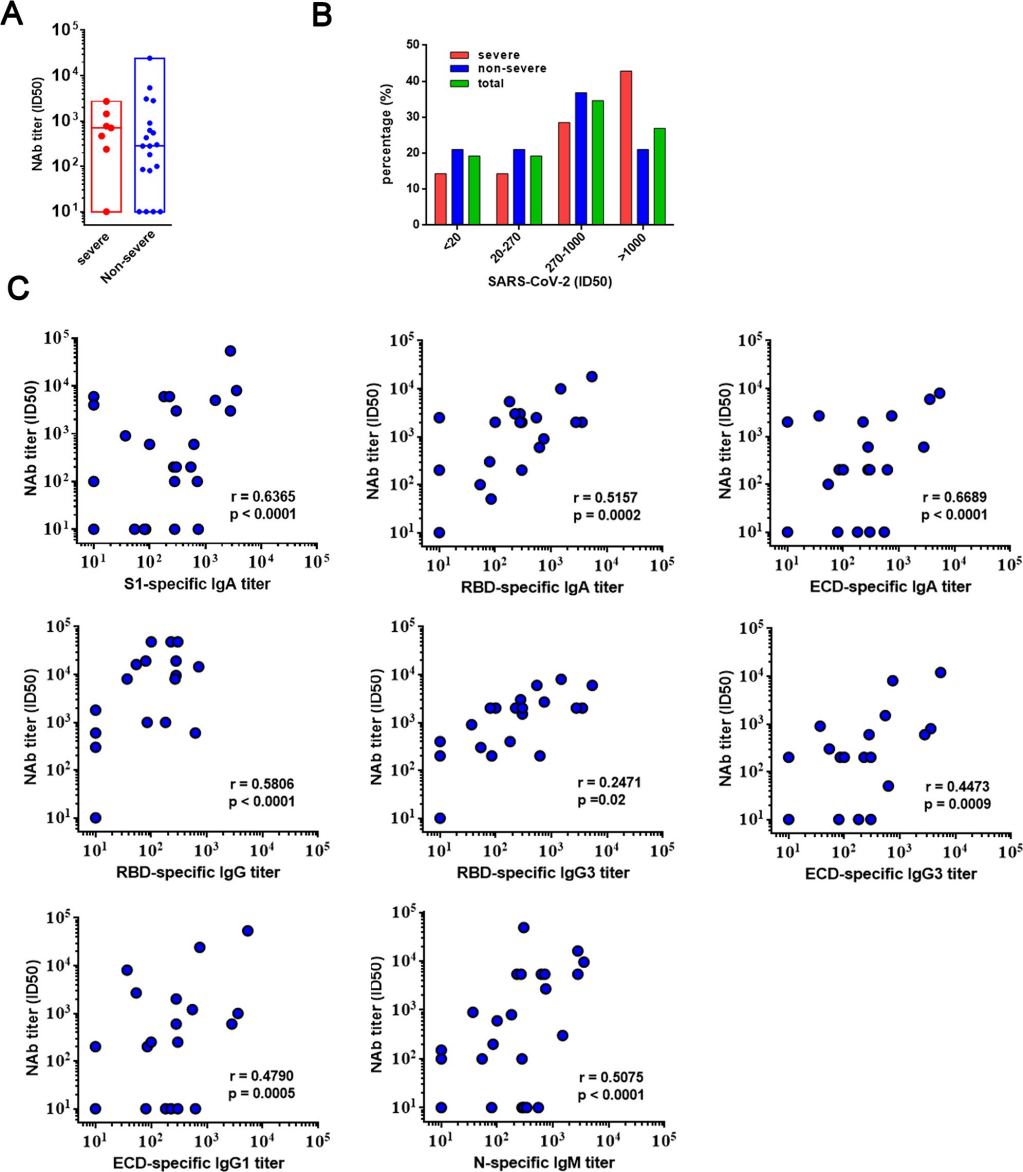
Neutralization activities of convalescent sera from COVID-19 recovered patients. **(A)** The neutralization titers (ID_50_) were determined between severe and non-severe patients. **(B)** The percentage of convalescent serum samples with undetectable neutralization titers (ID_50_ titer less than 20), low level of neutralization titers (ID_50_ titer between 20 to 270), moderate level of neutralization titers (ID_50_ titer between 270 to 1000), high level of neutralization titers (ID_50_ titer over 1000). **(C)** Correlation analysis of neutralization titers with the binding titers of S1-specific IgA, RBD-specific IgA, RBD-specific IgG, RBD-specific IgG3, N-specific IgM, ECD-specific IgA, ECD-specific IgG1 and ECD-specific IgG3.

The neutralization activities were subsequently determined by the pseudovirus microneutralization assay as previously described. 84.6% (22/26) of COVID-19 patients, including 85.7% (6/7) of the severe patients and 84.2% (16/19) of the non-severe patients, generated neutralization activities at the time point of discharge with a geometric mean titer of 274 **(Fig 5A and 5B)**. Meanwhile, 5/26 (19.23%) of patients elicited low levels of neutralization activities (ID_50_ titer between 20 to 270), while 9/26 (34.6%) of patients produced a moderate level of neutralization activities (ID_50_ titer between 271 to 1000), and 7/26 (26.92%) patients exhibited a potent level of neutralization activity (ID_50_ titer over 1000). There was no statistically significant difference in terms of neutralizing titers between the severe group (geometric titer, 772; IQR:470–1463) and non-severe group (geometric titer: 280; IQR, 82.5–584.5) (p = 0.18), despite the fact that the severe patients had considerably higher level of anti-SARS-CoV-2 specific binding antibody titers.

Using the antibody binding titers and neutralizing activities form the 19 non-severe patients, we discovered that the neutralizing antibody titers moderately correlated with the ECD-specific IgA responses (r = 0.6689, p<0.0001), S1-specific IgA response (r = 0.6365, p<0.0001), RBD-specific IgG responses (r = 0.5806, p<0.0001), RBD specific IgA responses (r = 0.5157, p = 0.0002), and NP-specific IgM responses (r = 0.5075, p<0.0001) **(Fig 5D)**. Furthermore, we also noticed that the ECD-specific IgG1 titer (r = 0.4790, p = 0.0005), ECD-specific IgG3 titer (r = 0.4473, p = 0.0009), and RBD-specific IgG3 titer (r = 0.2471, p = 0.0186) were weakly correlated with the serum neutralization activities. Additionally, in addition to IgG antibodies, IgA also greatly contributed to the anti-SARS-CoV-2 neutralizing activities. It is worth mentioning that in contrast to the correlation between the neutralizing activities and the binding antibody titers established in non-severe patients, we did not find any strong relationship between the neutralization activities and binding titers in the severe patients, suggesting a complex involvement of non-neutralizing antibodies during disease progression.

### The neutralizing activities were significantly declined concurrent with remarkedly diminished anti-RBD IgA responses

**Fig 6:**
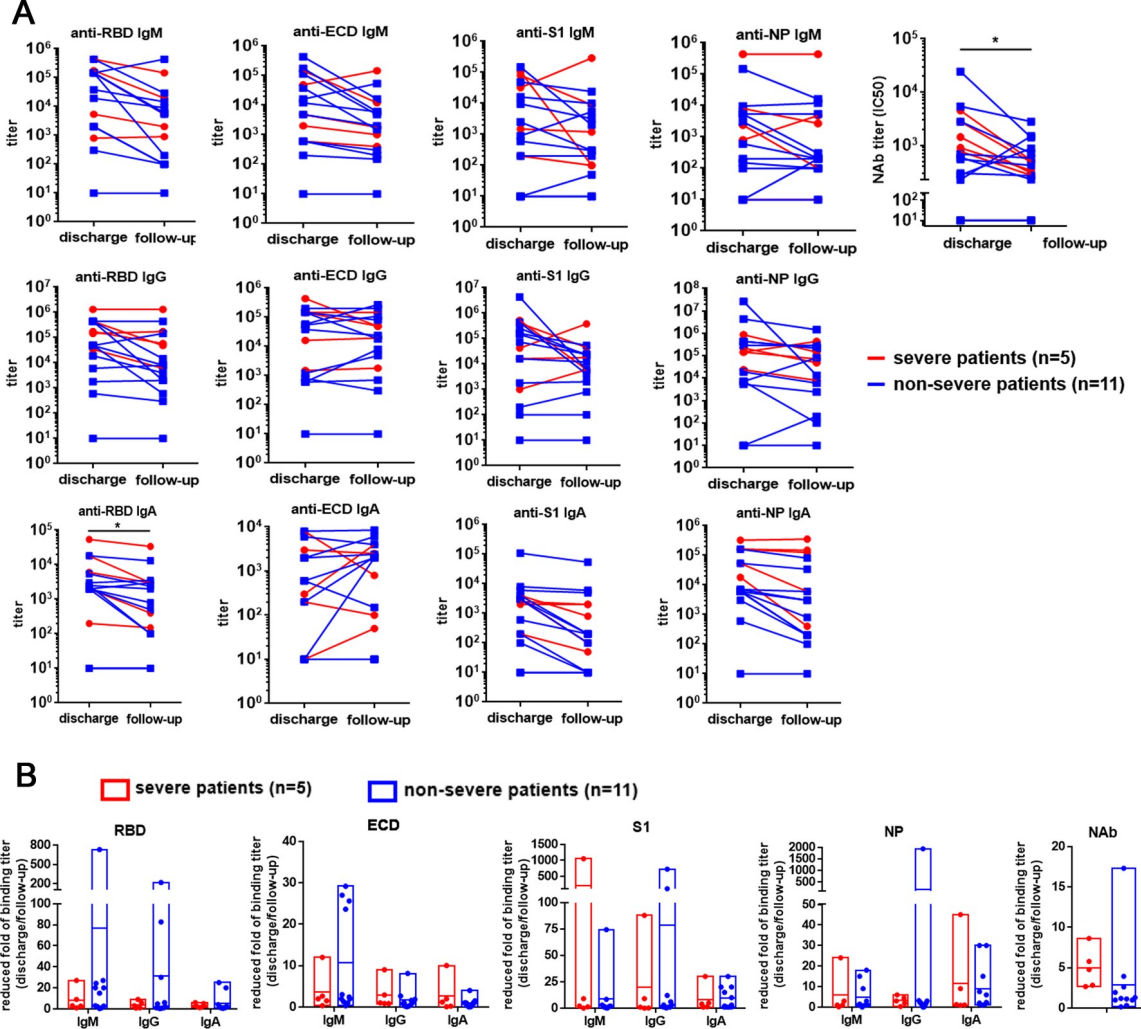
The dynamic humoral responses at the time point of hospital discharge and follow-up visit between 21 days and 28 days after discharge. **(A)** The comparison between the IgM, IgG, and IgA titer specific to RBD, ECD, S1, and NP protein and the neutralization activities at the time point of hospital discharge and follow up visit between 21 days and 28 days after discharge. **(B)** The reduced folds of IgM, IgG and IgA titer specific to RBD, ECD, S1, and NP protein and neutralization activities at the time point of hospital discharge and follow-up visit between 21 days and 28 days after discharge were analyzed between severe patients and non-severe patients.

Whether clinical recovered COVID-19 patients might be susceptible to SARS-CoV-2 re-infection remains questioned. Serum samples from sixteen COVID-19 patients (eleven non-severe and five severe patients) were collected at the time point of discharge and during follow-up visit between 21 and 28 days. Then, their neutralizing activities and binding antibody titers were further compared **(Fig 6A).** The neutralizing activities of serum obtained at the follow-up visit between 21 days and 28 days after discharge were significantly lower than those obtained at the time point of hospital discharge (p = 0.04, paired*-t* test), concurrent with significantly reduced anti-RBD IgA responses (p = 0.04, paired*-t* test), which might be responsible for the declining trend of neutralizing activities. Nevertheless, we did not observe a profoundly reduced IgM or IgG response targeting the NP or spike protein-related antigens. Additionally, the reduced fold of IgM, IgG and IgA binding titer and neutralization activities at the time point of hospital discharge and follow-up visit between 21 days and 28 days after discharge were also analyzed between severe patients and non-severe patients **(Fig 6B).** The severe patients and non-severe patients had comparable reduced fold of IgM, IgG, and IgA binding titer specific to RBD, ECD, S1, and NP protein and neutralization activities.

## Discussion

Using four recombinant SARS-CoV-2 related antigens, we were able to monitor the dynamic antibody responses with various specificities. We demonstrated that the kinetics profile of IgM or IgG responses specific for four antigens might be distinct in time and magnitude aspects. The ELISA assay was developed using RBD [5, 10], S1 [11], stabilized full-length spike protein [12], NP protein [13], combined RBD and NP protein (4), respectively, as serological assay, which is a feasible approach to analyze the SARS-CoV-2 specific humoral responses in COVID-19 patients and perform large-scale sero-epidemiology studies. Our current study suggested that combined detection of NP-specific IgM and ECD-specific IgG could greatly improve the sensitivity of serological assay especially during the first 2 weeks after symptom onset, compared to the mere inclusion of either the NP-specific antibody or RBD-specific antibody.

Despite of improved sensitivity of our ELISA assay, a small portion of non-severe COVID-19 patients was still identified as seronegative. This was consistent with recent findings. First, it is well-established that the non-severe COVID-19 patients generated significantly lower level of viral-specific humoral response compared to that in severe patients [14, 15], consistent with our results. Besides, asymptomatic SARS-CoV-2 carrier patients had a remarkably reduced level of virus specific IgG levels, compared to symptomatic group, accompanied by prolonged presence of viral RNA and remarkable reduction of IgG and neutralizing activities [16]. The differential pattern of specific humoral responses elicited by SARS-CoV-2 might be contributed by host factors such as age and host inflammatory responses [17, 18].

It is unclear whether clinically recovered patients acquire the protective immunity from re-infection. Limited information regarding the neutralization activities of the clinically recovered patients was available. In this study, we reported that 80.7% of convalescent sera had varying degrees of neutralization activities, and only a small portion of patients elicited a potent level of neutralization activity. Preliminary studies indicated that the major factor associated with the efficacy of convalescent plasma therapy is the neutralizing antibody titer of the convalescent plasma obtained from the donor [6, 19]. Our data also demonstrated the importance of prior selection of convalescent serum using neutralization assays. Additionally, the rapidly declined neutralizing activities in COVID-19 clinically recovered patients within 28 days after discharge, suggesting that the circulating anti-SARS-CoV-2 neutralizing antibodies might have a relatively short half-life. This finding was consistent with previous studies demonstrating that the humoral immunity rapidly waned over time in patients that recovered from SARS-CoV [20] and MERS-CoV [21] infections.

This study also revealed a pivotal role of mucosal immunity in humoral protection against SARS-CoV-2. First, we discovered that the neutralization activities were strongly associated with S1-specific IgA and ECD-specific IgA responses, and moderately correlated with RBD-specific IgA and IgG responses, suggesting that mucosal immunity might contribute greatly to viral neutralization. Even though our current temporal antibody profile did not include the IgA responses, previous studies demonstrated the presence of NP or RBD-specific IgA responses in the early phase during SARS-CoV-2 infection, whereas the systemic IgM and IgG responses occurred later [10, 13]. Of note, the RBD-specific IgA response declined sharply after hospital discharge, accompanied by the rapidly waned neutralization activities. Due to the relatively short half-life of IgA [22], our finding of rapidly declined RBD-specific IgA response is not surprising, which might be responsible for the clinical observation of prolonged viral RNA shedding in fecal samples [23] or re-occurrence of positive viral RNA in rectal swabs [24]. Furthermore, significantly higher level of IgA response was detected from the clinically recovered severe patients in comparison to non-severe patients. Consistent with our findings, vaccine-induced potent mucosal IgA was associated with lower level of viral load and reduced pulmonary pathological damages upon challenge with SARS-CoV [25]. The beneficial role of IgA during the COVID-19 disease course still requires thorough investigation. It is important to further analyze the magnitude of IgA responses between the survivor and non-survivor groups in a large cohort.

The antibody-dependent enhancement (ADE) phenomenon has been a major concern in viral infections [26–28]. Antibody specificity, concentration, neutralization ability, and isotype might define the ability of antibody to neutralize virions and protect the host or promote ADE and acute inflammation [29]. Previous animal studies of SARS-CoV indicated that the spike protein RBD region or HR2 domain-specific antibodies might exert a beneficial role in viral clearance, whereas the antibodies specific for NP and other S protein epitopes other than the RBD and HR2 domain might induce ADE and escalate acute inflammation [29, 30]. Besides, IgG isotype also controls the effector function, in which IgG1 and IgG3 engage FcrRIIa and FcrRIIb with high affinity leading to the possible occurrence of ADE. Our data showed that severe patients generated significantly higher level of antibody titers, especially for NP-specific IgG1 and RBD-specific IgG1 and IgG3 compared to the non-severe patients. Consistently, we did not identify any correlation between the binding antibody titers and the neutralizing activities from the convalescent sera of severe COVID-19 patients, suggesting that a large amount of non-neutralizing antibodies might have been present in the severe group. Whether such higher magnitude of NP-specific IgG1 or RBD-specific IgG1 and IgG3 contributed to the severity of COVID-19 disease progression remains to be determined.

Our data also have important implications for the current development of the COVID-19 vaccine. The majority of patients generated potent humoral responses recognizing spike protein-related antigens, including RBD, S1 and ECD proteins, implying their high immunogenicity makes them vaccine candidates. Additionally, our correlation analyses showed that the neutralizing activities were strongly correlated with ECD-specific IgA and S1-specific IgA responses, compared to RBD-specific IgA responses. Several human neutralizing monoclonal antibodies (mAbs) isolated from convalescent COVID-19 patients were not exclusively targeted at RBD region. For example, the epitope of a potent neutralizing mAb, 4A8, is within N terminal of spike protein [31], while other mAbs targeted at cryptic epitopes on spike trimeric interface [32]. Collectively, our study and others suggested that the antibodies targeting at diverse domains of the spike protein might greatly contribute to higher neutralization activities. Consequently, the inclusion of the full-length spike protein might be ideal to elicit humoral responses targeting to the major neutralizing targets. Moreover, the IgG subclass responses in COVID-19 patients were skewed toward IgG1 and IgG3, and induction of optimal antibody isotypes such as IgA and certain IgG subclasses such as IgG2 might be desired in vaccine studies of SARS-CoV-2.

Our study also has some limitations. First, we only included a small number of recovered COVID-19 patients and did not have the deceased COVID-19 patients. Second, the cytokines and antigen-specific cellular responses were not serially monitored, which could facilitate our understanding of innate and adaptive immune responses during COVID-19 disease. Thirdly, the mucosal IgA responses such as the IgA responses in saliva warrant further investigation.

In conclusion, our study demonstrated that the majority of COVID-19 patients generated a distinct profile of immune response against NP and spike protein-related antigens in both time and magnitude aspects. Therefore, combining NP and ECD as detecting antigens could further enhance the sensitivity of the serological assay. Furthermore, 80.7% of the convalescent sea from COVID-19 patients displayed varying levels of neutralization activities against SARS-CoV-2, which correlated with S1-specific and ECD-specific IgA responses in non-severe patients. A rapid decline in these neutralizing activities was observed, accompanied by a sharply reduced RBD-specific IgA response. Our comprehensive, longitudinal analysis provides clues for the optimization of future serological testing, antibody-based intervention, and vaccine design.

## Supporting information

S1 Fig Dynamic IgM or IgG responses specific to NP, RBD, S1, and ECD antigens for four severe COVID-19 patients (P7 to P10) and sixteen non-severe COVID-19 patients (P11 to P26) over time since symptom onset, determined by ELISA assay. (TIF)

S1 Table Laboratory findings and drug treatment of severe and non-severe COVID-19 patients. (DOCX)

## References

[1] Organization WH. Coronavirus disease 2019 (COVID-19) situation Report-91. 2020.

[2] Guan WJ, Ni ZY, Hu Y, Liang WH, Ou CQ, He JX, et al Clinical Characteristics of Coronavirus Disease 2019 in China. N Engl J Med. 2020. 10.1056/NEJMoa2002032 PMC7092819 32109013

[3] Yongchen Z, Shen H, Wang X, Shi X, Li Y, Yan J, et al Different longitudinal patterns of nucleic acid and serology testing results based on disease severity of COVID-19 patients. Emerg Microbes Infect. 2020:1–14. 10.1080/22221751.2020.1756699 PMC7241531 32306864

[4] To KK, Tsang OT, Leung WS, Tam AR, Wu TC, Lung DC, et al Temporal profiles of viral load in posterior oropharyngeal saliva samples and serum antibody responses during infection by SARS-CoV-2: an observational cohort study. Lancet Infect Dis. 2020. 10.1016/S1473-3099(20)30196-1 PMC7158907 32213337

[5] Perera RA, Mok CK, Tsang OT, Lv H, Ko RL, Wu NC, et al Serological assays for severe acute respiratory syndrome coronavirus 2 (SARS-CoV-2), March 2020. Euro Surveill. 2020;25(16). 10.2807/1560-7917.ES.2020.25.16.2000421 PMC7189648 32347204

[6] Shen C, Wang Z, Zhao F, Yang Y, Li J, Yuan J, et al Treatment of 5 Critically Ill Patients With COVID-19 With Convalescent Plasma. JAMA. 2020. 10.1001/jama.2020.4783 PMC7101507 32219428

[7] Duan K, Liu B, Li C, Zhang H, Yu T, Qu J, et al Effectiveness of convalescent plasma therapy in severe COVID-19 patients. Proc Natl Acad Sci U S A. 2020. 10.1073/pnas.2004168117 PMC7196837 32253318

[8] China NHCotPsRo. Chinese management guideline for COVID-19 (version 6.0). Feb 19, 2020.

[9] Nie J, Li Q, Wu J, Zhao C, Hao H, Liu H, et al Establishment and validation of a pseudovirus neutralization assay for SARS-CoV-2. Emerg Microbes Infect. 2020;9(1):680–6. 10.1080/22221751.2020.1743767 32207377 PMC7144318

[10] Zhao J, Yuan Q, Wang H, Liu W, Liao X, Su Y, et al Antibody responses to SARS-CoV-2 in patients of novel coronavirus disease 2019. Clin Infect Dis. 2020. 10.1093/cid/ciaa344 PMC7184337 32221519

[11] Okba NMA, Muller MA, Li W, Wang C, GeurtsvanKessel CH, Corman VM, et al Severe Acute Respiratory Syndrome Coronavirus 2-Specific Antibody Responses in Coronavirus Disease 2019 Patients. Emerg Infect Dis. 2020;26(7). 10.3201/eid2607.200841 PMC7323511 32267220

[12] Amanat F, Stadlbauer D, Strohmeier S, Nguyen THO, Chromikova V, McMahon M, et al A serological assay to detect SARS-CoV-2 seroconversion in humans. Nat Med. 2020. 10.1038/s41591-020-0913-5 PMC8183627 32398876

[13] Guo L, Ren L, Yang S, Xiao M, Chang, Yang F, et al Profiling Early Humoral Response to Diagnose Novel Coronavirus Disease (COVID-19). Clin Infect Dis. 2020. 10.1093/cid/ciaa310 PMC7184472 32198501

[14] Juanjuan Zhao Jr. QY, Haiyan Wang, Wei Liu, Xuejiao Liao, Yingying Su, Xin Wang, Jing Yuan, Tingdong Li, Jinxiu Li, Shen Qian, Congming Hong, Fuxiang Wang, Yingxia Liu, Zhaoqin Wang, Qing He, Zhiyong Li, Bin He, Tianying Zhang, Shengxiang Ge, Lei Liu, Jun Zhang, Ningshao Xia, Zheng Zhang. Antibody responses to SARS-CoV-2 in patients of novel coronavirus disease 2019. medRxiv.2020.03.02.20030189. 10.1093/cid/ciaa344 PMC7184337 32221519

[15] Liu ZL, Liu Y, Wan LG, Xiang TX, Le AP, Liu P, et al Antibody profiles in mild and severe cases of COVID-19. Clin Chem. 2020. 10.1093/clinchem/hvaa137 PMC7314168 32521002

[16] Long QX, Tang XJ, Shi QL, Li Q, Deng HJ, Yuan J, et al Clinical and immunological assessment of asymptomatic SARS-CoV-2 infections. Nat Med. 2020. 10.1038/s41591-020-0965-6 32555424

[17] Zhang X, Tan Y, Ling Y, Lu G, Liu F, Yi Z, et al Viral and host factors related to the clinical outcome of COVID-19. Nature. 2020. 10.1038/s41586-020-2355-0 32434211

[18] Wu F, Wang A, Liu M, Wang Q, Chen J, Xia S, et al Neutralizing antibody responses to SARS-CoV-2 in a COVID-19 recovered patient cohort and their implications. medRxiv. 2020.

[19] Zhang B, Liu S, Tan T, Huang W, Dong Y, Chen L, et al Treatment with convalescent plasma for critically ill patients with SARS-CoV-2 infection. Chest. 2020. 10.1016/j.chest.2020.03.039 PMC7195335 32243945

[20] Cao WC, Liu W, Zhang PH, Zhang F, Richardus JH. Disappearance of antibodies to SARS-associated coronavirus after recovery. N Engl J Med. 2007;357(11):1162–3. 10.1056/NEJMc070348 17855683

[21] Arabi YM, Hajeer AH, Luke T, Raviprakash K, Balkhy H, Johani S, et al Feasibility of Using Convalescent Plasma Immunotherapy for MERS-CoV Infection, Saudi Arabia. Emerg Infect Dis. 2016;22(9):1554–61. 10.3201/eid2209.151164 27532807 PMC4994343

[22] Stockert RJ, Kressner MS, Collins JC, Sternlieb I, Morell AG. IgA interaction with the asialoglycoprotein receptor. Proc Natl Acad Sci U S A. 1982;79(20):6229–31. 10.1073/pnas.79.20.6229 6292896 PMC347093

[23] Wu Y, Guo C, Tang L, Hong Z, Zhou J, Dong X, et al Prolonged presence of SARS-CoV-2 viral RNA in faecal samples. Lancet Gastroenterol Hepatol. 2020;5(5):434–5. 10.1016/S2468-1253(20)30083-2 32199469 PMC7158584

[24] Zhang BL S,; Dong Y; Zhang L.; Zhong Q.; Zou Y.; Zhang S. Positive rectal swabs in young patients recovered from coronavirus disease 2019 (COVID-19). J Infection. 2020 10.1016/j.jinf.2020.04.023 32335176 PMC7177113

[25] Du L, Zhao G, Lin Y, Sui H, Chan C, Ma S, et al Intranasal vaccination of recombinant adeno-associated virus encoding receptor-binding domain of severe acute respiratory syndrome coronavirus (SARS-CoV) spike protein induces strong mucosal immune responses and provides long-term protection against SARS-CoV infection. J Immunol. 2008;180(2):948–56. 10.4049/jimmunol.180.2.948 18178835 PMC2603051

[26] Iwasaki A, Yang Y. The potential danger of suboptimal antibody responses in COVID-19. Nat Rev Immunol. 2020. 10.1038/s41577-020-0321-6 PMC7187142 32317716

[27] Liu L, Wei Q, Lin Q, Fang J, Wang H, Kwok H, et al Anti-spike IgG causes severe acute lung injury by skewing macrophage responses during acute SARS-CoV infection. JCI Insight. 2019;4(4). 10.1172/jci.insight.123158 PMC6478436 30830861

[28] Katzelnick LC, Gresh L, Halloran ME, Mercado JC, Kuan G, Gordon A, et al Antibody-dependent enhancement of severe dengue disease in humans. Science. 2017;358(6365):929–32. 10.1126/science.aan6836 29097492 PMC5858873

[29] Wang Q, Zhang L, Kuwahara K, Li L, Liu Z, Li T, et al Immunodominant SARS Coronavirus Epitopes in Humans Elicited both Enhancing and Neutralizing Effects on Infection in Non-human Primates. ACS Infect Dis. 2016;2(5):361–76. 10.1021/acsinfecdis.6b00006 27627203 PMC7075522

[30] Yasui F, Kai C, Kitabatake M, Inoue S, Yoneda M, Yokochi S, et al Prior immunization with severe acute respiratory syndrome (SARS)-associated coronavirus (SARS-CoV) nucleocapsid protein causes severe pneumonia in mice infected with SARS-CoV. J Immunol. 2008;181(9):6337–48. 10.4049/jimmunol.181.9.6337 18941225

[31] Chi X, Yan R, Zhang J, Zhang G, Zhang Y, Hao M, et al A neutralizing human antibody binds to the N-terminal domain of the Spike protein of SARS-CoV-2. Science. 2020. 10.1126/science.abc6952 PMC7319273 32571838

[32] Wu Y, Li C, Xia S, Tian X, Kong Y, Wang Z, et al Identification of Human Single-Domain Antibodies against SARS-CoV-2. Cell Host Microbe. 2020;27(6):891–8 e5. 10.1016/j.chom.2020.04.023 32413276 PMC7224157

