# Supplementary figures and images for: A comprehensive, longitudinal analysis of humoral responses specific to four recombinant antigens of SARS-CoV-2 in severe and non-severe COVID-19 patients
Source: PLoS Pathog. 2020 Sep 10;16(9):e1008796. doi: 10.1371/journal.ppat.1008796 (PMC7482996; doi:10.1371/journal.ppat.1008796)

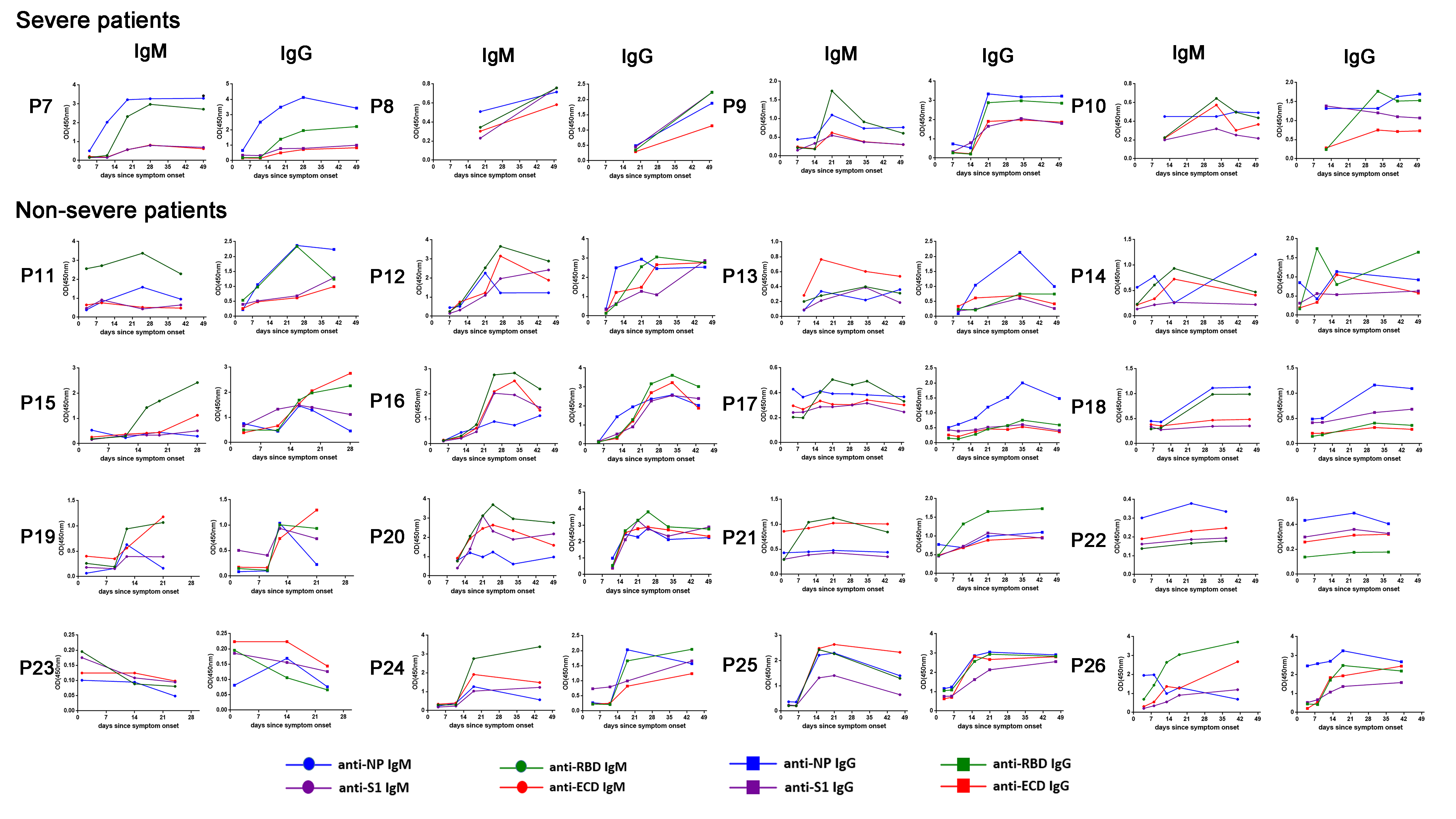

Supplement: S1 Fig — (TIF) [file ppat.1008796.s001.tif]
