# Supplementary material for: A comprehensive, longitudinal analysis of humoral responses specific to four recombinant antigens of SARS-CoV-2 in severe and non-severe COVID-19 patients
Source: PLoS Pathog. 2020 Sep 10;16(9):e1008796. doi: 10.1371/journal.ppat.1008796 (PMC7482996; doi:10.1371/journal.ppat.1008796)
Supplement: S1 Table — (DOCX) [file ppat.1008796.s002.docx]

**A Comprehensive, longitudinal analysis of humoral responses specific to four recombinant antigens of SARS-CoV-2 in severe and non-severe COVID-19 patients**

**S1 Table. Laboratory findings and drug treatment of severe and non-severe COVID-19 patients.**

| Variables (n [%] or median [IQR]) | All patients (n=26) | severe (n=7) | Non-severe (n=19) | p value* |
| --- | --- | --- | --- | --- |
| **Lymphocyte subsets** |  |  |  |  |
| CD45+ Lymphocyte(×10^9^/L) | 1308.0 (1032.0, 1954.0) | 1068.0 (1033.0, 1217.0) | 1458.0 (1062.0, 2156.0) | 0.010 |
| CD3+ total T cells (%) | 74.6 (60.5, 78.3) | 58.8 (58.1, 73.6) | 75.7 (67.2, 78.0) | 0.101 |
| CD3+ total T cells(×10^6^/L) | 915.5 (627.3, 1342.8) | 677.0 (603.0, 844.0) | 1173.0 (662.0, 1693.0) | 0.007 |
| CD4+ T cells(×10^6^/L) | 524.5 (372.8, 657.8) | 394.0 (376.0, 575.0) | 563.0 (374.0, 696.0) | 0.182 |
| CD4+/CD45+ | 38.5 (26.5, 47.3) | 38.0 (36.0, 50.0) | 39.0 (25.5, 46.5) | 0.209 |
| CD8+/CD45+ | 27.0 (20.5, 31.8) | 20.0 (16.5, 24) | 29.0 (23.0, 33.0) | 0.055 |
| CD8+ T cells(×10^9^/L) | 299.0 (221.0, 562.0) | 242.0 (194.5, 280.5) | 485.0 (224.5, 580.5) | 0.005 |
| CD4+CD8+/CD45+ | 0.09 (0, 0.17) | 0.15 (0.05, 0.23) | 0.07 (0, 0.16) | 0.153 |
| CD4+CD8+ T cells(×10^9^/L) | 1.0 (0, 3.5) | 2.0 (0.5, 3) | 1 (0, 3) | 0.466 |
| Th/Ts (CD4+CD8+) | 1.5 (0.9, 2.4) | 2.3 (1.6, 3.2) | 1.2 (0.8, 1.8) | 0.058 |
| CD16/56+ NK cells (%) | 12.7 (7.4, 23.0) | 24.8 (13.4, 31.9) | 11.4 (6.9, 15.6) | 0.048 |
| CD16/56 NK cells(×10^6^/L) | 181.0 (116.0, 263.0) | 258.0 (160.0, 330.0) | 172.0 (111.0, 220.0) | 0.137 |
| CD19+ B cells (%) | 12.9 (8.5, 14.4) | 9.5 (7.5, 13.3) | 13.4 (9.1, 14.7) | 0.163 |
| CD19+ B cells (×10^6^/L) | 136.0(12.0, 200.0) | 112.0 (91.0, 135.0) | 171.0 (118.0, 263.0) | 0.007 |
| **Blood routine** |  |  |  |  |
| Platelet (×10^9^/L) | 232.0 (181.0, 271.0) | 180.0 (149.0, 222.0) | 246.0 (193.0, 310.0) | 0.016 |
| ESR (mm/h) | 15.5 (6.3, 34.3) | 21.0 (12.0, 39.0) | 10.0 (5.5, 30.0) | 0.333 |
| PT (S) | 11.9 (11.3, 12.5) | 11.5 (10.7, 13.7) | 11.9 (11.4, 12.2) | 0.445 |
| APTT (S) | 29.4 (26.5, 35.8) | 33.5 (28.5, 37.1) | 28.8 (26.5, 33.5) | 0.212 |
| PCT (ng/ml) | 0.02 (0.016, 0.041) | 0.018 (0.015, 0.024) | 0.022 (0.016, 0.043) | 0.054 |
| D-dimer (mg/L) | 0.22 (0.19, 0.37) | 0.19 (0.19, 0.26) | 0.3 (0.2, 0.4) | 0.173 |
| Troponin (ng/mL) | 0.04 (0.03, 0.05) | 0.035 (0.015, 0.04) | 0.04 (0.04, 0.05) | 0.061 |
| Albumin (g/L) | 43.6 (36.2, 46.3) | 44.5 (42.9, 44.7) | 41.9 (35.5, 47.8) | 0.272 |
| Creatinine (μmol/L) | 51.5 (43.5, 63.5) | 46.0 (41.5, 47.0) | 57.0 (46.7, 70.4) | 0.016 |
| ALT (U/L) | 32.7 (16.7, 54.7) | 46.1 (14.9, 73.4) | 31.4 (19.3, 51.2) | 0.222 |
| LDH (IU/L) | 226.0 (197.0, 296.0) | 303.0 (213.0, 317.0) | 224.0 (191.0, 259.0) | 0.253 |
| serum ferritin (μmol/L) | 13.7 (10.5, 16.5) | 8.2 (6.7, 13.7) | 14.9 (11.9, 16.6) | 0.044 |
| **Drug treatment** |  |  |  |  |
| hormone | 12 (46.2) | 7 (100) | 5 (26.3) | 0.001 |
| Interferon atomization | 25 (96.2) | 7 (100) | 18 (94.7) | 1.000 |
| Lopinavir and Ritonavir | 16 (61.5) | 6 (85.7) | 10 (52.6) | 0.190 |
| Immunoglobulin | 12 (46.2) | 6 (85.7) | 6 (31.6) | 0.026 |
| Arbidol | 16 (61.5) | 5 (71.4) | 11 (57.9) | 0.668 |
| Darunavir | 11 (42.3) | 3 (42.9) | 8 (42.1) | 1.000 |
| Thymosin | 3 (11.5) | 1 (14.3) | 2 (10.5) | 1.000 |
| Ribavirin | 5 (19.2) | 0 (0) | 5 (26.3) | 0.278 |

IQR, interquartile range; ESR, erythrocyte sedimentation rate; PT, prothrombin time; APTT, activated partial thromboplastin time; PCT, procalcitonin; ALT, alanine transaminase; LDH, lactate dehydrogenase. *p value refers to the statistical difference between severe and non-severe group.
